# Investigation of the safety of Radium-223 chloride in combination with external beam radiotherapy for bone metastases of prostate cancer

**DOI:** 10.1093/jrr/rraf002

**Published:** 2025-02-08

**Authors:** Soichi Makino, Kazunari Miyazawa, Yoji Katsuoka, Takeru Ooe, Ken Aikawa, Akira Segawa, Hiroshi Kobayashi

**Affiliations:** Department of Radiology (Radiation therapy), Shinkuki General Hospital, 418-1 Kami-Hayami, Kuki, Saitama 346-8530, Japan; Department of Radiology, Showa General Hospital, 8-1-1 Hanakoganei, Kodaira, Tokyo 187-8510, Japan; Yamato Tsukimino Jin Clinic, 9-10-1 Chuorinkan, Yamato, Kanagawa 242-0007, Japan; Department of Medical Technology, Shinkuki General Hospital, 418-1 Kami-Hayami, Kuki, Saitama 346-8530, Japan; Department of Urology, Shinkuki General Hospital, 418-1 Kami-Hayami, Kuki, Saitama 346-8530, Japan; Department of Urology, Saiseikai Kazo Hospital, 1680 Kamitakayanagi, Kazo, Saitama 347-0101, Japan; Department of Urology, Saiseikai Kazo Hospital, 1680 Kamitakayanagi, Kazo, Saitama 347-0101, Japan

**Keywords:** Ra-223, external beam radiotherapy, castration-resistant prostate cancer, bone metastases, safety

## Abstract

This study aimed to investigate the safety of combining radium-223 chloride (Ra-223) therapy with external beam radiation therapy (EBRT) for patients with multiple bone metastases from castration-resistant prostate cancer (CRPC), including lesions requiring urgent treatment such as those causing neurological symptoms due to spinal cord compression. We retrospectively analyzed data from patients with CRPC and bone metastases treated with Ra-223 therapy at our hospital between September 1, 2018, and December 31, 2023. Adverse events were evaluated using the Common Terminology Criteria for Adverse Events version 4.0. Of the 23 patients referred, data from 17 were included; 8 received concurrent Ra-223 therapy and EBRT, whereas others received only Ra-223 therapy. The median follow-up period was 20 months. Grade (G) 2 or higher adverse events occurred in seven patients (41.2%), and G 3 or higher in 2 (11.7%). None of the patients who received EBRT with fields involving the gastrointestinal tract experienced diarrhea, constipation, bleeding, perforation, or obstruction. Ra-223 therapy with EBRT did not increase adverse events compared with studies of Ra-223 therapy without EBRT. One case of G 5 Pneumocystis carinii pneumonia, likely because of steroid use for neurological symptoms and the patient’s underlying diabetes mellitus, was noted. The effects of EBRT cannot be entirely excluded, so minimizing field size and dose is recommended when combining Ra-223 therapy and EBRT. Our findings indicate that concurrent Ra-223 therapy and EBRT could be safe for managing patients with symptomatic bone metastases and castration-resistant prostate cancer who require specialized treatment, provided sufficient attention is given to the field and the prescribed dose.

## INTRODUCTION

The incidence of prostate cancer in Japan is alarmingly high [1]. Various treatment options for localized prostate cancer are available, each with favorable outcomes [1–6]; however, some patients may relapse and develop castration-resistant prostate cancer (CRPC), which is challenging to treat despite subsequent systemic therapy. CRPC, often accompanied by symptomatic bone metastases, severely affects the quality of life and shortens the lifespan. Radium-223 chloride (Ra-223), a bone-targeting radiopharmaceutical, is a promising alternative. The Alpharadin in Symptomatic Prostate Cancer (ALSYMPCA) trial demonstrated the effectiveness of Ra-223 therapy in pain relief, improving survival (3.6 months), and delaying the progression of bone-related complications (7.5 months) in patients with CRPC and bone metastases [7]. Despite these promising results, the optimal timing of Ra-223 therapy and its safety in combination with other treatments remains unclear. External beam radiation therapy (EBRT) or surgery to manage spinal cord compression has been recommended for patients with CRPC receiving Ra-223 therapy. However, as verified using strontium-89 chloride (Sr-89) therapy [8], specific safety data for concurrent administration are lacking [9, 10]. In clinical practice, some lesions worsen during Ra-223 therapy despite an overall good therapeutic response. This scenario suggests the need for EBRT or surgery, along with Ra-223 therapy. Reports indicate that the combination of Ra-223 therapy and EBRT is safe, with no increased hematologic toxicity, and that pelvic irradiation in combination with Ra-223 therapy is also well tolerated [11]. However, data on the safety of this combination therapy remains limited. This study aimed to investigate the safety of Ra-223 therapy combined with EBRT for multiple bone metastases, including lesions requiring urgent treatment, such as neurological symptoms owing to spinal cord compression.

## MATERIALS AND METHODS

Patients diagnosed with CRPC and bone metastases who were referred to receive Ra-223 therapy at the radiology (treatment) department of our hospital between September 1, 2018, and December 31, 2023, were evaluated for Ra-223 therapy eligibility based on established criteria [7]. These criteria included no visceral metastases, bone scintigraphy showing renewed accumulation consistent with bone metastases, adequate blood counts (neutrophils >1500/μl, platelets >100 000/μl, hemoglobin >10.0 g/dL before the first dose), preserved bone marrow function, no administration of abiraterone, and no severe hepatic or renal dysfunction. Patients with pre-existing spinal cord compression symptoms due to vertebral metastases were excluded from this study. Patients presenting with spinal cord compression symptoms due to bone metastases at the initial presentation are first treated with EBRT or surgery, even if they are referred to Ra-223 therapy. Ra-223 therapy was administered only when symptom relief was achieved. The patients received Ra-223 therapy (administered intravenously at a dose of 55 kBq/kg at 4-week intervals for a maximum of six cycles). Baseline clinical and imaging characteristics (age, Eastern Cooperative Oncology Group performance status [PS] score, initial prostate-specific antigen [iPSA] level [ng/ml], initial Gleason score, initial N stage, presence of super bone scan on bone scintigraphy [SBS] [12] before Ra-223 therapy administration, use of bone-modifying agents, previous abiraterone or enzalutamide use, previous chemotherapy, and previous EBRT) were collected. SBS refers to widespread metastasis throughout the bones. Concurrent EBRT was defined as EBRT delivered to a bone lesion within 4 weeks of initiating Ra-223 therapy. The primary endpoint was the rate of Grade (G) 2 or higher adverse events (AEs), whereas the secondary endpoints were the rates of treatment completion, survival, and symptom palliation. Based on a report indicating that prognosis was better in cases completing six doses of Ra-223 than in those who did not, even though it was a single-arm analysis [13], treatment completion was defined as receiving six doses of Ra-223. Survival was analyzed according to SBS before the Ra-223 therapy administration. Treatment efficacy was evaluated by a radiologist using diagnostic imaging. Ra-223 therapy was discontinued in cases of disease progression, and other treatment modalities were recommended. AEs were evaluated using the Common Terminology Criteria for Adverse Events version 4.0. Survival rates were calculated using the Kaplan–Meier method, and significance tests were performed using the log-rank method. Statistical analyses were performed using the Statistical Package for Social Sciences version 22.0 for Windows (IBM Japan, Tokyo). A *P*-value <0.05 was considered statistically significant.

This retrospective observational study was approved by our institutional review board (approval no. 108) in compliance with the Declaration of Helsinki and the ethical guidelines for life sciences and medical research involving human subjects. The board waived the need for informed consent for data collection, as this was a retrospective study.

## RESULTS

### Patient baseline characteristics

During this period, 23 patients were referred to the department for Ra-223 therapy, and 19 patients received treatment with Ra-223 therapy. Four patients did not receive Ra-223 therapy for the following reasons: hemoglobin levels below 10.0 g/dL before the first dose, liver metastases, and declined Ra-223 therapy. Of the patients who received Ra-223 therapy, 17 had completed or discontinued treatment as of the data cut-off date; therefore, 17 patients were included in the analysis. The data of six patients (26.1%) were not analyzed for the following reasons: two (8.7%) were on treatment at the time of analysis, two (8.7%) had hemoglobin levels of <10.0 g/dL before the first dose, one (4.3%) had liver metastases, and one (4.3%) declined Ra-223 therapy. The baseline characteristics of the patients are summarized in Table 1. The median age at treatment initiation was 76 years (range, 66–91 years). PS ranged from 0 to 2, with 10 patients (58.8%) having a PS of 1. The median PSA level before Ra-223 therapy was 36.9 ng/ml (range, 7.3–1917 ng/ml). The median follow-up period was 20 months (range, 1–55 months).

**Table 1:** Patients’ baseline characteristics

Characteristics		All patients	Ra-223 + EBRT	Ra-223 alone
Age (years)	Median (range)	76 (66–91)	77 (72–90)	76 (66–91)
PS	0	4	1	3
	1	10	5	5
	2	3	2	1
iPSA (ng/ml)	Median(range)	39.0 (4.6–9040)	52.5 (24–4423)	30.6 (4.6–9040)
Gleason score	7	5	2	3
	8	0	0	0
	9	7	3	4
	10	1	0	1
	Unknown	4	3	1
Initial N stage	0	9	5	4
	1	8	3	5
SBS	Yes	6	4	2
	No	11	4	7
Use of bone modifying agents	Yes	15	8	7
	No	2	0	2
Previous abiraterone and/or enzalutamide	Yes	11	6	5
	No	6	2	4
Previous chemotherapy	Yes	4	2	2
	No	13	6	7
Previous EBRT	Yes	9	4	5
	No	8	4	4

PS, Eastern Cooperative Oncology Group performance status; iPSA, initial prostate-specific antigen; SBS, Super bone scan; Ra-223, Radium-223 chloride; EBRT, external beam radiotherapy.

### Treatment and AEs

The overall incidence of G2 or higher AEs was 7 (41.2%), and that of G3 or higher AEs was 2 (11.7%). The most frequent AE was anemia (G2, two patients; G3, one patient), followed by delirium (G2, one patient (5.9%)), vomiting (G2, one patient (5.9%)), fatigue (G2, one patient (5.9%)), and a serious fungal infection, Pneumocystis carinii pneumonia (PCP) (G5, one patient (5.9%)). The incidence of AEs above G2 or higher in the concurrent EBRT group was 4 (57.1%) (three cases of anemia and one case of PCP). Details of the distribution of ≥ G2 AEs can be found in Table 2. No SSEs were observed in any case during the follow-up period.

**Table 2:** Adverse events

	Grade	All patients	Ra-223 + EBRT	Ra-223 alone
Cases		7	4	3
Anemia	2	2	2	0
	3	1	1	0
Delirium	2	1	0	1
Vomiting	2	1	0	1
Fatigue	2	1	0	1
PCP	5	1	1	0

Adverse events were evaluated using Common Terminology Criteria for Adverse Events version 4.0.

PCP, pneumocystis carinii pneumonia; EBRT, external beam radiotherapy.

The overall treatment completion rate was 52.9% (50.0% in the concurrent EBRT group and 55.6% in the Ra-223 alone group). The reasons for discontinuation were disease progression (each for first, second, and third administrations), patient preference (first administration), fatigue (first administration), nausea (first administration), PCP (third administration), and severe anemia (fifth administration). Concurrent EBRT was performed in 47.1% (8/17) of the patients. In 35.3% (6/17) of the cases, the field included the gastrointestinal tract (small or large intestines). The median dose administered was 30 Gy (range, 8–42 Gy). The timing of EBRT was as follows: after the first administration in three patients, after the second in two patients, after the fourth in two patients, and after completion all in one patient. The reasons for concurrent EBRT were spinal cord compression symptoms (four cases), urinary retention due to the primary tumor (two cases), and rib pain (two cases). The fields of concurrent EBRT were the vertebrae (three cases), vertebrae and lymph nodes (one case), ribs (two cases), prostate (one case), and prostate and pelvic lymph nodes (one case). Spinal cord compression symptoms were reduced, and urinary retention was relieved in all six patients. Reduction in rib pain could not be evaluated because opioids were administered. None of the patients who underwent concurrent EBRT with a field encompassing the intestinal tract experienced diarrhea, constipation, bleeding, perforation, or obstruction.

### Survival outcomes

**Fig. 1:**
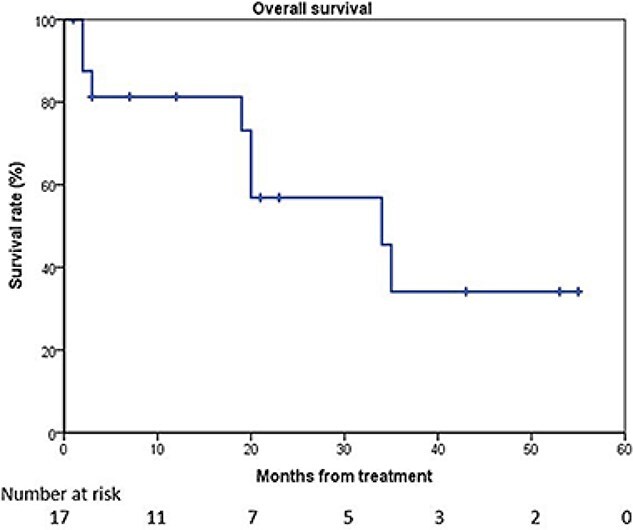
Kaplan–Meier estimate of overall survival. The 2-year overall survival rate was 56.9%.

**Fig. 2:**
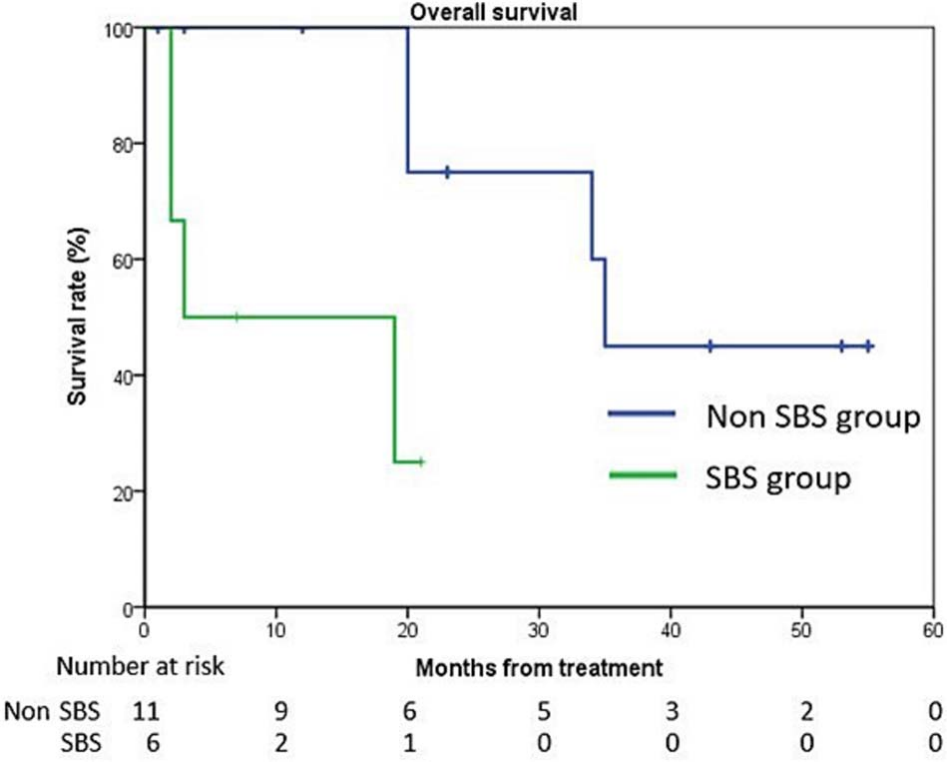
Kaplan–Meier estimate of overall survival in the SBS subgroup (SBS group, non- SBS group). The 2-year overall survival rate of the non-SBS group was 75.0% (*P* = 0.010). SBS, super bone scan on bone scintigraphy.

The 2-year overall survival rate was 56.9% (Fig. 1). The presence of SBS on pretreatment bone scintigraphy was a significantly poor prognostic factor for survival (Fig. 2) (log-rank test, *P* = 0.010).

## DISCUSSION

This study investigated the safety of concurrent EBRT in patients with CRPC and symptomatic bone metastases. Our results indicate that combining Ra-223 therapy with EBRT did not increase AEs.

The overall incidence of G3 or higher AEs in our study (11.7%) was comparable to that reported in the ALSYMPCA trial (23.3% of G3 or higher AEs), which evaluated Ra-223 monotherapy [7]. The rate of G3 or higher AEs in our study was also lower than or comparable to the rates reported in retrospective studies in Japan with similar patient numbers [14, 15]. This finding may indicate that concurrent EBRT does not significantly increase the risk of severe AEs. The ALSYMPCA trial evaluated patients who received EBRT; however, the rate of patients who received concurrent EBRT was very low, and safety was not evaluated [16]. Although the ADRRAD trial used the prostate as the target organ and did not target the bone or lymph nodes, concurrent EBRT was used, and its safety was suggested. The most informative study for our clinical question was a phase II trial of concurrent EBRT after randomization in 64 patients with CRPC experiencing painful bone metastases who were deemed to require EBRT [10]. This study is reliable because it is a prospective study that specifically addresses clinical bone metastasis pain.

However, the reason for introducing EBRT was different from that for patients who required EBRT during the clinical course in our study. While that study suggested the safety of combining Ra-223 therapy with EBRT, the patients in this study were only those who required EBRT because of bone pain. Notably, our study objective was slightly different because we examined the safety of concurrent EBRT in patients in whom symptoms requiring treatment, such as spinal cord compression, necessitating EBRT for symptom relief. We encountered a wide range of cases in clinical practice. For patients with reduced bone scintigraphic accumulation in all bone lesions, reduced alkaline phosphatase, and resolution or reduction of clinical symptoms, Ra-223 therapy was continued. For patients with increased bone scintigraphic accumulation, elevated alkaline phosphatase levels, and worsening clinical symptoms, treatment should be discontinued, and other options should be considered. For patients with increased bone scintigraphic accumulation in one area and decreased accumulation in others, we added EBRT aggressively to the site of exacerbation, especially if bone metastasis exacerbation presents with neurological symptoms. A phase II trial is also being conducted in Japan for oligometastatic CRPC, with progression-free survival and death from any cause as primary endpoints [17]. The results of this phase II trial may provide a reference for the safety of EBRT in combination with Ra-223 therapy but may not be the ultimate answer to safety concerns. This trial involved patients with 1–3 bone metastases who were considered to have a good prognosis, whereas ours involved real clinical cases where EBRT was an added necessity due to the onset of some symptoms. Although the aim of this trial is to evaluate the curative potential of the treatment, we aimed to evaluate the safety of our medical treatment, which we believe is justified from a radiobiological perspective and considered necessary,

Each AE was studied microscopically, starting with hematological toxicity. Hematopoietic function is dependent on red bone marrow, and its impairment shows a dose-dependent relationship with radiation exposure above a threshold dose. The risk of radiation-induced hematopoietic suppression increases with higher radiation doses and larger treatment volumes [18]. Hematological toxicity, particularly myelosuppression, is a potential concern with combined Ra-223 therapy and EBRT, owing to their effects on the bone marrow. However, the incidence of anemia of G2 or higher severity in our study was relatively low (three patients, 17.6%). This finding aligns with observations from the ALSYMPCA trial, which reported a lower incidence of hematologic toxicity with Ra-223 therapy than with other bone-targeting radiopharmaceuticals, such as Sr-89 [7]. This result is supported by modeling studies of the dose to the bone marrow, which showed that the equivalent dose to healthy bone marrow is much lower with Ra-223 than with Sr-89 [19]. This difference can be attributed to the shorter range of alpha particles in Ra-223 than that of the beta particles emitted by Sr-89, resulting in a lower radiation dose to the healthy bone marrow. The increased frequency of hematologic toxicity in the ADRRAD trial compared with the ALSYMPCA trial may be because 93.3% of patients with ADRRAD received doxorubicin before treatment [11]. Similarly, the small number of patients in our patient population who received chemotherapy before Ra-223 therapy may explain the lower incidence of hematologic toxicities. In this study, three cases of anemia and one case of PCP occurred in the concurrent EBRT group; therefore, an association between concurrent EBRT and reduced bone marrow function cannot be ruled out. However, all four cases were SBS, which may be related to reduced bone marrow function due to bone metastases in the first place [20]. If bone marrow function was significantly compromised, leukopenia might be expected to develop, but it did not. One possible explanation is that steroids were administered in all four cases, as these were situations in which EBRT was added to manage neurological symptoms caused by bone metastases [21].

Regarding gastrointestinal toxicity, we did not observe an increased risk of gastrointestinal AEs in patients who received concurrent EBRT in a field encompassing the intestinal tract. The lower prescribed dose in our study (median 30 Gy) compared to the ADRRAD (approximately 74 Gy) likely explains this difference. Additionally, all patients in our case group received a lower intestinal dose in their treatment plans. We re-examined the radiotherapy plans of the patients in this case group. In all cases where the irradiated field also included the gastrointestinal tract, we found that the treatment plan for each patient involved over three beams from the posterior, right, and left sides or intensity-modulated radiation therapy with suppressed intestinal doses.

Finally, bone-related events were examined. Fractures at non-metastatic sites have been reported in several patients in an ERA-223 randomized controlled trial of Ra-223 therapy in combination with abiraterone. This report is more common in the abiraterone group and is suspected to be related to the bone density-reducing effects of luteinizing hormone-releasing hormones and steroids. This was likely due to the lowering effect of the therapy on bone mineral density. In addition, there have been reports of radiation-induced fractures in patients with EBRT >50 Gy, likely owing to blood vessel changes causing weakening of the bone tissue [22] and resulting in fractures. However, the mechanisms underlying pathological fractures differ. Radiation-induced fragility arises from two primary mechanisms: direct damage to osteoblasts, osteocytes, and osteoclasts caused by radiotherapy and indirect damage from radiation-induced bone hemorrhage, which disrupts the supply of essential nutrients to bone cells, leading to further bone loss. The latter requires more attention in combination with EBRT. Although no pathological fractures were observed in this case group, further long-term safety verification is needed for bone-related events because radiation-induced fractures are more likely to occur over 2 years [23].

The completion rate of 52.9% in our study was slightly lower than the 57% rate in the Japanese domestic phase II trial [24]. Our treatment completion rates were also lower than those reported in single-center retrospective studies in Japan with similar patient numbers [14, 25]. However, this completion rate is reasonable considering the high average age of the patients in our retrospective study, the presence of SBS, and the absence of the Hawthorne effect. In this study, 41.2% of the patients had SBS, and their prognoses were poorer than those of other groups, as reported by other researchers [26]. The present study included many patients with SBS due to Ra-223 therapy provided by the radiotherapy department. Moreover, >50% of the patients had previously received EBRT in the department or were referred to EBRT. Specifically, 82.4% of the patients received EBRT in our hospital during all periods. However, survival could not be evaluated based on these results because of the small sample size.

The case of PIP in G5 are discussed separately. In this case, the patient presented with spinal symptoms due to bone metastases in the lumbar spine at the initial presentation. Radiotherapy (30 Gy/10 Fr) was performed on Th12-L5 for symptomatic relief. After symptoms improved, Ra-223 therapy was administered. After two courses of Ra-223 therapy, radiotherapy was performed to relieve ureteral obstruction due to internal iliac lymph node metastases and ureteral obstruction due to primary enlargement. Betamethasone 4 mg/day was initially administered and tapered to 2 mg/day, but aspiration pneumonia developed at this point [27]. After one week of treatment with levofloxacin 500 mg/day, the pneumonia was mildly relieved; however, it recurred after treatment cessation. The patient was administered levofloxacin again, but the symptoms did not improve. Blood tests showed elevated β-D glucan and a sputum culture was performed, which tentatively confirmed PIP. Although the influence of Ra-223 therapy cannot be entirely ruled out, the primary cause appears to be prolonged high-dose betamethasone. Severe diabetes mellitus in this patient may also have increased susceptibility to infection. Regarding EBRT, this patient had the highest prescribed dose of 42 Gy/21 Fr in our cases, with the largest irradiated volume, and most of the field included the pelvic bone (Supplementary Fig. S1-1.1-2). However, such infections did not occur in other cases with comparable fields (Supplementary Fig. S2).

Notably, in this study, symptoms were relieved in all patients following EBRT. In our hospital, the patients were evaluated twice a month during Ra-223 therapy, and imaging, such as magnetic resonance imaging and bone scintigraphy, was immediately accessible because of active collaboration with radiotherapy technicians. This allowed for the early detection of issues such as spinal cord compression because of bone metastases, which may have contributed to symptom relief [28]. However, no definitive evidence supports this hypothesis.

This study had some limitations. First, the small sample size restricts the generalizability of our findings, and the retrospective nature of the study introduces potential bias. Second, the Bone Scan Index (BSI) is commonly used to quantify metastatic bone disease and predict biochemical response to Ra-223 therapy [29]; however, BSI data were not collected in this study. Instead, we used the SBS, which can be criticized for not being quantitative. Despite these limitations, the use of EBRT with Ra-223 therapy for the management of spinal cord compression symptoms is noteworthy. In clinical practice, some lesions worsen during Ra-223 therapy despite an overall good therapeutic response. If the lesion causes spinal cord compression symptoms, EBRT or surgery is performed, whereas Ra-223 therapy is discontinued. We commonly encounter cases in which other lesions worsen when Ra-223 therapy is ceased, and the patient is ultimately unable to complete the six courses. Considering the impact of completing six courses of Ra-223 therapy on prognosis, the study’s findings are noteworthy [30]. Treatment of spinal cord compression symptoms, if present, should be prioritized because the analgesic effect of Ra-223 therapy may mask the pain caused by spinal cord compression, and emergency treatment is required to prevent paralysis. However, we suggest that this may not be the case if EBRT, which treats spinal cord compression symptoms, is performed simultaneously with Ra-223 therapy.

In conclusion, our findings suggest that concurrent Ra-223 therapy and EBRT is a potentially safe approach for managing patients with CRPC who experience symptomatic bone metastases and require special treatment. However, careful patient selection, appropriate EBRT field design, and dose optimization are crucial for minimizing toxicity risks. Therefore, more attention should be paid to anemia due to bone marrow dysfunction in patients with SBS. Further studies with larger prospective cohorts are required to establish the safety and efficacy of this combination therapy.

## Supplementary Material

Supplementary_Figure_1_1_rraf002

Supplementary_Figure_1_2_rraf002

Supplementary_Figure_2_rraf002 Reproduced from Makino S, Miyazawa K, Kastuoka Y *et al.* Physical and social pain relief by external beam radiotherapy and radium-223 dichloride without opioids: A case report. *Jpn J Urol Surg* 2023;36:411–16.
