# Supplementary figures and images for: Investigation of the safety of Radium-223 chloride in combination with external beam radiotherapy for bone metastases of prostate cancer
Source: J Radiat Res. 2025 Feb 8;66(2):137–43. doi: 10.1093/jrr/rraf002 (PMC11932343; doi:10.1093/jrr/rraf002)

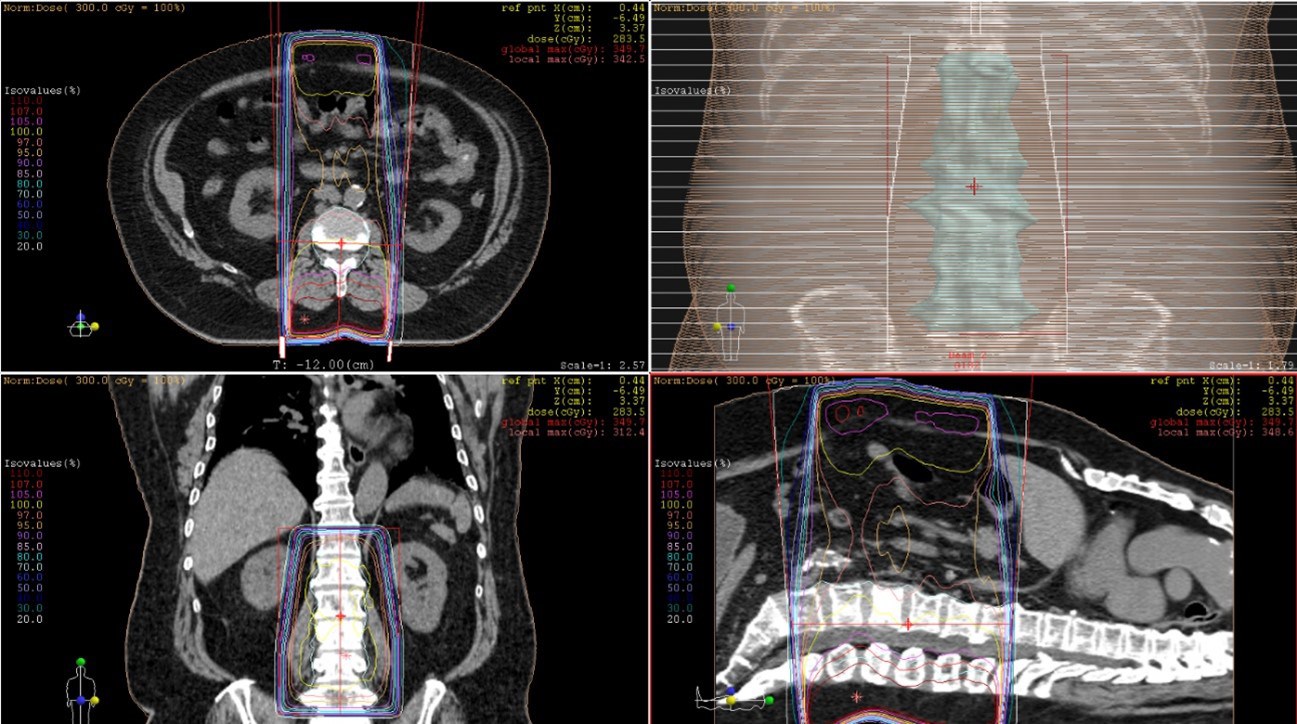

Supplement: Supplementary_Figure_1_1_rraf002 [file supplementary_figure_1_1_rraf002.jpeg]

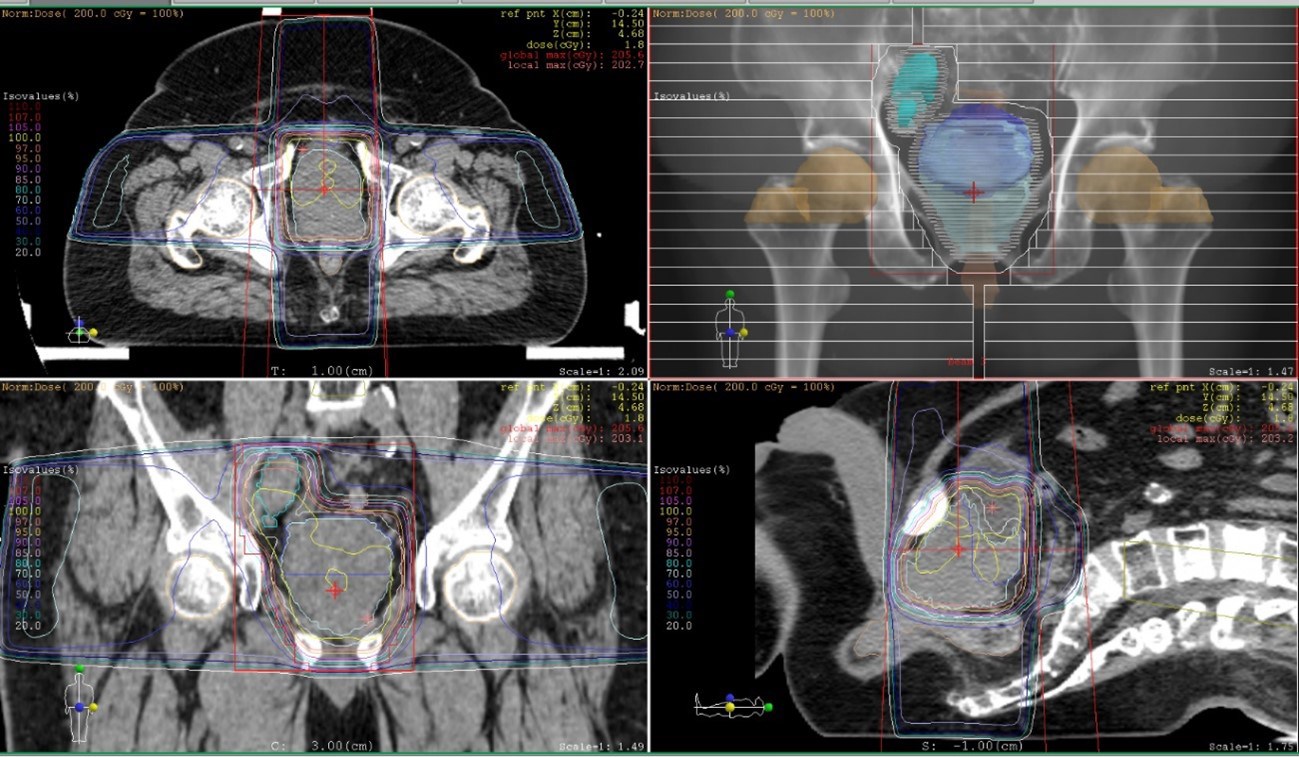

Supplement: Supplementary_Figure_1_2_rraf002 [file supplementary_figure_1_2_rraf002.jpeg]

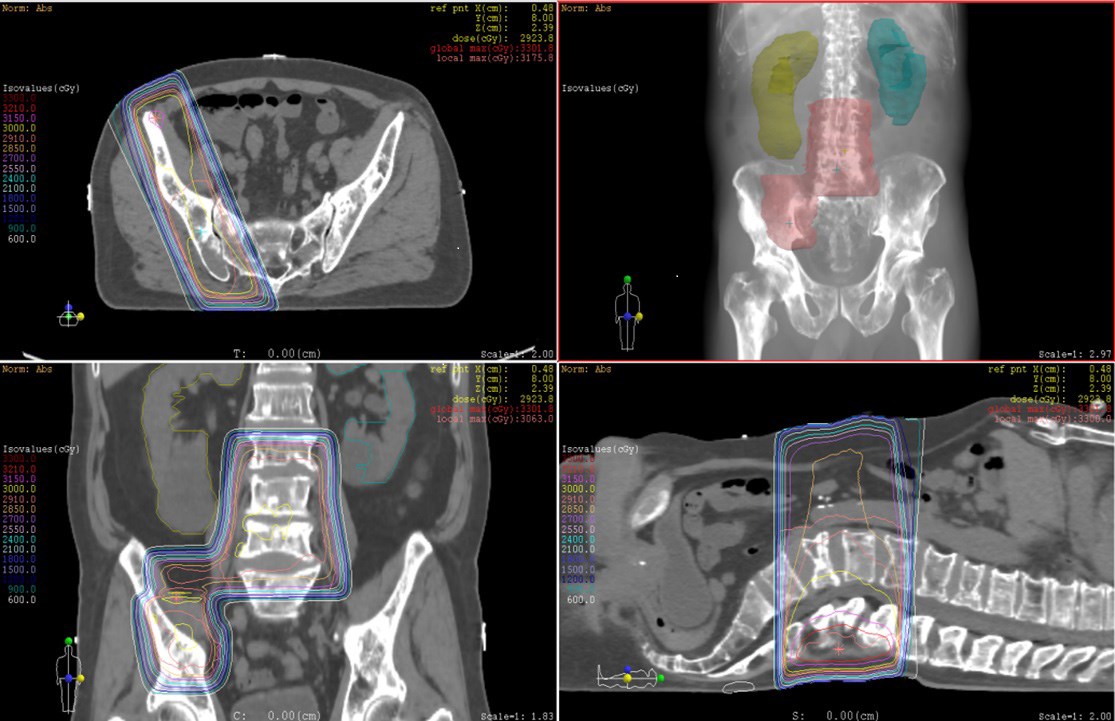

Supplement: Supplementary_Figure_2_rraf002 — Reproduced from Makino S, Miyazawa K, Kastuoka Y et al. Physical and social pain relief by external beam radiotherapy and radium-223 dichloride without opioids: A case report. Jpn J Urol Surg 2023;36:411–16. [file supplementary_figure_2_rraf002.jpeg]
